# Initial vaccination and revaccination with Type I PRRS 94881 MLV reduces viral load and infection with porcine reproductive and respiratory syndrome virus

**DOI:** 10.1186/s40813-018-0096-3

**Published:** 2018-08-20

**Authors:** Jeremy Kroll, Michael Piontkowski, Christian Kraft, Teresa Coll, Oliver Gomez-Duran

**Affiliations:** 1 Department of Research and Development, Boehringer Ingelheim Animal Health Inc, 2412 South Loop Drive, Ames, IA 50010 USA; 2 Boehringer Ingelheim Animal Health, 2621 N. Belt Hwy, St Joseph, MO 64506 USA; 3 Boehringer Ingelheim Veterinary Research Center GmbH & Co. KG, Bemeroder Straβe 31, 30559 Hannover, Germany; 4 Boehringer Ingelheim Vetmedica GmbH, Binger Straβe 173, 55216 Ingelheim am Rhein, Germany

**Keywords:** Porcine reproductive respiratory syndrome (PRRS), Viral RNA load, Revaccination, PRRS 94881 MLV

## Abstract

**Background:**

Porcine reproductive and respiratory syndrome (PRRS) causes respiratory distress in pigs, reproductive failure in breeding-age gilts and sows, and can have devastating economic consequences in domestic herds. Several PRRS vaccines are available commercially. This study compared the effectiveness of single-vaccination and revaccination schedules using the PRRS 94881 Type I modified live virus (MLV) vaccine ReproCyc® PRRS EU with no vaccination (challenge control) in protecting against a PRRS virus (PRRSV) challenge in non-pregnant gilts.

**Results:**

Data were available from 48 gilts across three groups: a challenge control group (*n* = 16), which received no vaccination; a revaccination group (*n* = 16), which received ReproCyc® PRRS EU on Days 0 and 56; and a single vaccination group (*n* = 16), which received ReproCyc® PRRS EU on Day 56. All gilts were PRRSV RNA-negative (based on reverse transcription and quantitative polymerase chain reaction [RT-qPCR]) and PRRSV seronegative (based on enzyme-linked immunosorbent assay [ELISA]) at Day 0. All gilts were challenged with PRRSV strain 190136 on Day 91.

Viral RNA loads in both vaccination groups were significantly reduced compared with the challenge control group on Days 98 (*P* < 0.0001) and 101 (P < 0.0001), indicating that vaccinated gilts were better able to respond to challenge than unvaccinated gilts. At all timepoints following challenge, mean viral RNA load and the percentage of PRRSV RNA-positive gilts were numerically higher in the single-vaccination group than in the revaccination group; these differences were statistically significant on Day 101 (*P* = 0.0434). Furthermore, viremia levels after challenge were significantly lower in the revaccination group than in the single-vaccination group based on median area under the curve (AUC) values for viral RNA load from Day 91 to Day 112, suggesting that revaccinated gilts had better protection from viral infection than gilts who received a single vaccination. Protection from viremia did not correlate with the proportion of seropositive gilts on Day 91. In the single-vaccination group, 94% of pigs were seropositive on Day 91 compared with 56% in the revaccination group. Vaccination was well tolerated and no safety concerns were identified.

**Conclusions:**

Both single-vaccination and revaccination with ReproCyc® PRRS EU were effective in reducing PRRSV viremia post-challenge. These findings have important implications for herd management as both the single-vaccination and revaccination schedules protect against PRRSV challenge, with revaccination appearing to provide better protection from viremia than single vaccination.

## Background

Porcine reproductive and respiratory syndrome (PRRS) is a viral disease that causes respiratory distress, reproductive failure and increases mortality in pigs [1–3]. PRRS was initially reported in North America in the late 1980s and in Europe in the 1990s and has since spread rapidly [3, 4]. It is now known to affect swine worldwide [3] and is responsible for devastating economic losses in domestic herds [2, 5].

PRRS is caused by the PRRS virus (PRRSV), a positive strand RNA virus belonging to the *Arteriviridae* family [2, 3]. Historically, PRRSV has been divided into two main genotypes: a Type I European (EU) genotype and a Type II North American (NA) genotype, which are antigenically different [3] and are now considered to be different species [6]. A variant of the NA genotype designated as highly pathogenic PRRSV recently emerged in Asia and has been shown to cause severe disease [3].

PRRSV is transmitted through direct contact with body fluids such as saliva, urine, milk and semen (naturally and through artificial insemination), and through indirect means such as contamination via needles, fomites, farm personnel, vehicles, insect vectors, and airborne mechanisms including aerosolization [3, 4, 7–9]. The virus can therefore spread quickly through a herd, especially if the animals are kept in close proximity, and may also spread to nearby herds [3].

Although infection can be asymptomatic, clinical features occur due to acute viremia in adult or weaned pigs and transmission of the virus across the placenta to developing fetuses. Concomitant infection with other pathogenic microorganisms may also occur. Clinical features typically include systemic effects (such as loss of appetite, weight loss, fever, diarrhea, and lethargy), and effects on the respiratory system (e.g. labored breathing and respiratory distress) and on reproduction (e.g. premature farrowing and abortion, still birth, pre-weaning death, mummified fetuses, and variable size and weak-born piglets) [3, 10]. The severity of disease depends on many factors, including the strain of virus, immune status of the animal, the age of the infected animal and herd husbandry [3, 9]. Effective PRRSV prevention and control strategies such as pig flow, gilt acclimation and herd management are therefore essential, especially as pigs are still contagious for a period following recovery from clinical disease [11–13].

Vaccines against PRRS are available but are usually used to reduce clinical signs and control disease rather than to prevent infection due to the difficulty in protecting from heterologous PRRSV strains [5]. At least 20 different vaccines are commercially available worldwide, comprising modified live viruses (MLVs) and killed PRRS viruses [5, 14]. Of these, MLV vaccines are considered most effective in protecting pigs from circulating PRRSV, provided the MLV is antigenically similar to the circulating strain [14].

PRRS MLV vaccines have been shown to significantly reduce fetal infection, to improve pig health and reproductive performance, and to enhance the body weight and survival rate at weaning of piglets born to vaccinated gilts compared with piglets born to non-vaccinated gilts [5, 15, 16]. In growing pigs, PRRS MLV vaccines can reduce viremia, and improve respiratory signs and growth performance [17, 18].

Despite these positive results, questions over the efficacy and safety of current PRRS MLV vaccines have been raised, including concerns over virus genotype-specific protection, interference with other swine vaccines, and reversion to virulence [5]. In addition, there is disagreement within the swine health field on the most appropriate vaccination schedule; it is unclear whether multiple injections (repeated at 8-weekly intervals) are better than a single injection at limiting clinical signs and providing immunity against subsequent infection. There are continuing efforts to develop new PRRS vaccines that are safe, highly immunogenic, and confer broad protection across PRRSV strains [19, 20].

ReproCyc® PRRS EU is a PRRS MLV which has previously been shown to be safe and efficacious in several clinical trials [21–24]. This study evaluated whether revaccination with ReproCyc® PRRS EU using a two-dose vaccination scheme would result in protection from viremia equivalent to or greater than with a single-dose vaccination.

## Results

Data were available from 48 non-pregnant gilts across three groups: a challenge control group (*n* = 16), which received no vaccination; a revaccination group (*n* = 16), which received ReproCyc® PRRS EU on Days 0 and 56; and a single-vaccination group (*n* = 16), which received ReproCyc® PRRS EU on Day 56 only. All gilts were PRRSV RNA-negative (as determined by reverse transcription and quantitative polymerase chain reaction [RT-qPCR]) and PRRSV seronegative (as determined by enzyme-linked immunosorbent assay [ELISA]) at the start of the study.

### Viremia and viral RNA load

**Fig. 1:**
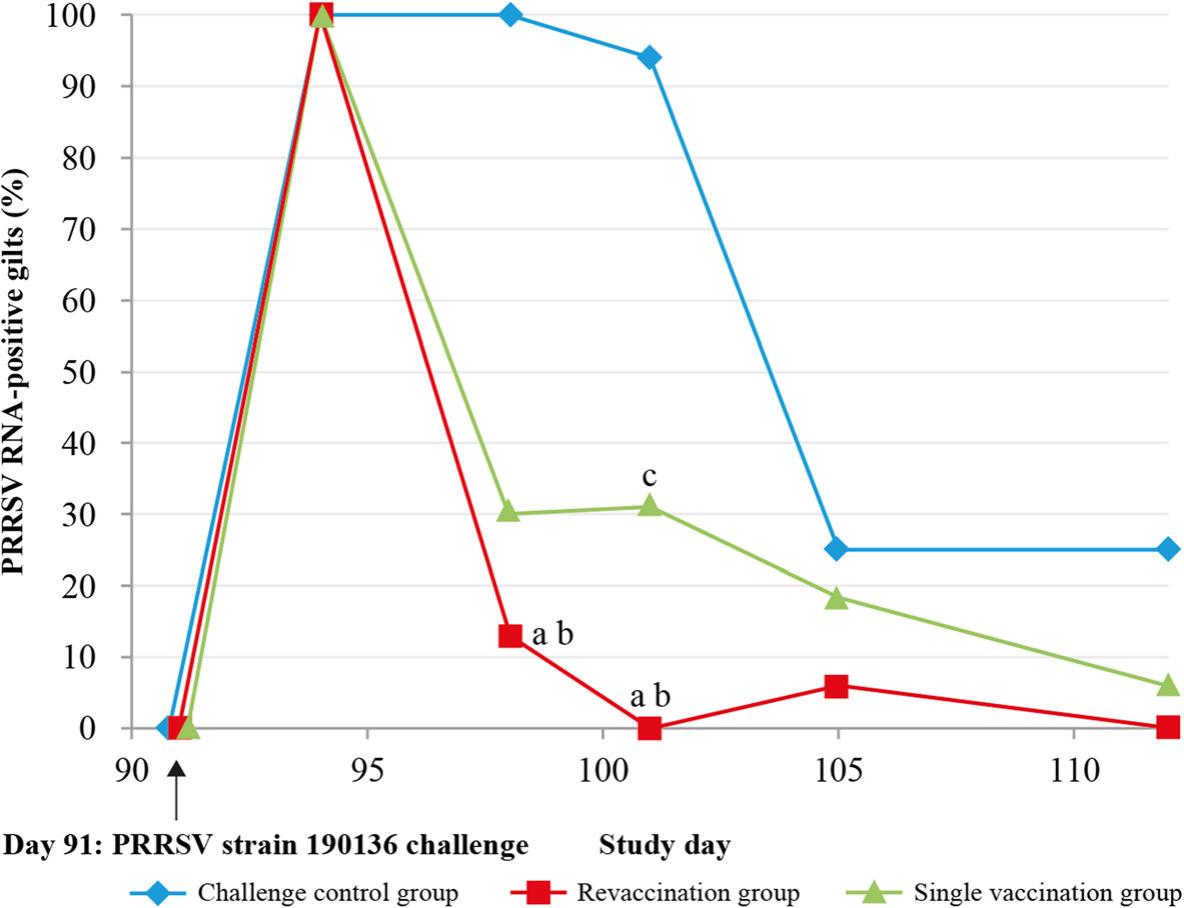
Percentage of PRRSV RNA-positive gilts after challenge with PRRSV strain 190,136. Viral load was assessed using RT-qPCR. All gilts were challenged with PRRSV strain 190136 on Day 91. Prior to challenge (Day 0–91), 0% of gilts were PRRSV RNA-positive. On Day 94, 100% tested positive for PRRSV RNA. The percentage of PRRSV RNA-positive gilts then declined sharply in both the revaccination and single-vaccination groups. By Day 98 both vaccination groups had significantly lower percentages of PRRSV RNA-positive gilts than the challenge control group (*P* < 0.0001). Challenge control group (*n* = 16); Revaccination group (*n* = 16); Single-vaccination group (*n* = 16). ^a^P < 0.0001 for challenge control vs revaccination group; ^b^P < 0.001 for challenge control vs single-vaccination group; ^c^P < 0.05 for revaccination group vs single-vaccination group

The percentage of gilts that were positive for PRRSV RNA from day of challenge (Day 91) to Day 112 based on RT-qPCR is shown in Fig. 1. After the challenge on Day 91, all gilts tested positive for PRRSV RNA by Day 94. The percentage of PRRSV RNA-positive gilts then declined sharply in both the revaccination and single-vaccination groups, and by Day 98 both vaccination groups had significantly lower percentages of PRRSV RNA-positive gilts than the challenge control group (*P* < 0.0001). After Day 101 (10 days after challenge), the percentage of PRRSV RNA-positive gilts in the challenge control group fell considerably and there were no significant differences in the percentages of PRRSV RNA-positive gilts between the vaccine groups and the challenge control group by Day 105.

At all timepoints following challenge, the percentage of PRRSV RNA-positive gilts was numerically higher in the single-vaccination group than in the revaccination group, and this difference was statistically significant on Day 101 (*P* = 0.0434).

**Fig. 2:**
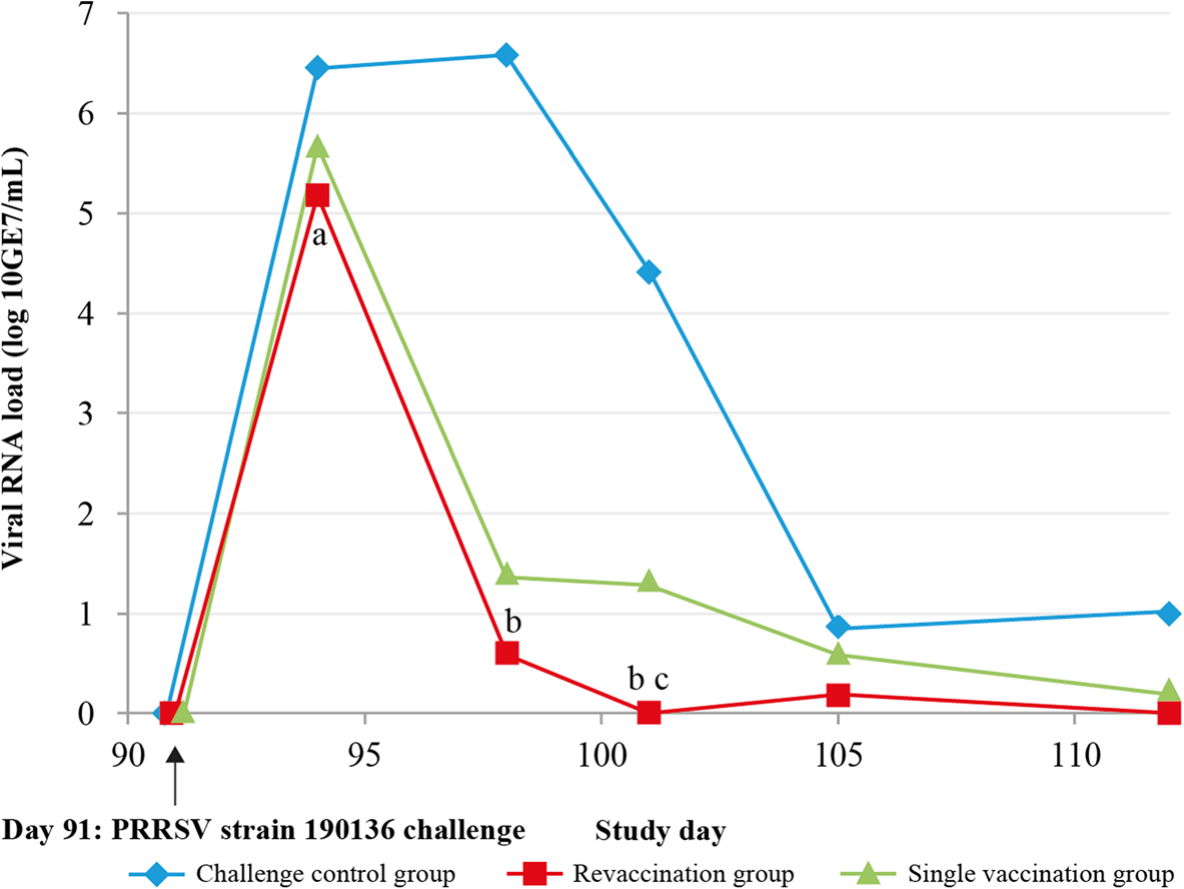
PRRSV viral RNA load in gilts after challenge with PRRSV strain 190136**.** Serum PRRS viral RNA load was assessed using RT-qPCR. Viral RNA load was baseline in all groups from Day 0–91. All gilts were challenged with PRRSV strain 190,136 on Day 91. The viral RNA loads of both vaccination groups were significantly reduced compared with the challenge control group on Days 98 (P < 0.0001) and Day 101 (*P* < 0.0001). Challenge control group (*n* = 16); Revaccination group (*n* = 16); Single-vaccination group (*n* = 16). ^a^P < 0.001 for challenge control vs revaccination group; ^b^*P* < 0.0001 for challenge control vs revaccination or single-vaccination group; ^c^P < 0.05 for revaccination group vs single-vaccination group

Serum PRRS viral RNA load based on RT-qPCR was baseline in all groups from study initiation to Day 91, indicating that vaccinated gilts were free of PRRSV up until the point of challenge on Day 91 (Fig. 2). Viral RNA load increased sharply between challenge and Day 94 in all groups. However, the revaccination group had significantly lower median viral RNA loads compared with the challenge control group on Day 94 (*P* = 0.0007). The duration of viremia post-challenge was shorter in the revaccination and single-vaccination groups compared with the challenge control group. On Day 98, all challenge control gilts were still viremic whereas only two revaccinated gilts and five single-vaccinated gilts were PRRSV RNA-positive. The viral RNA loads of both vaccination groups were significantly reduced compared with the challenge control group on Day 98 (*P* < 0.0001 for both) and Day 101 (*P* < 0.0001 for both).

As with the percentage of PRRSV RNA-positive gilts, mean viral RNA load at all timepoints following challenge was numerically higher in the single-vaccination group than in the revaccination group, and this difference was statistically significant on Day 101 (*P* = 0.0434).

The median AUC values for viral RNA load from Day 91 to Day 112 (AUC_91–112_) are shown in Table 1. Median AUC_91–112_ values were significantly lower for both vaccination groups compared with the challenge control group (both *P* < 0.0001) and the median AUC_91–112_ for the revaccination group was significantly lower than for the single-vaccination group (*P* = 0.0029).

**Table 1:** AUC median values from Day 91 to Day 112 in gilt groups, after challenge with PRRSV strain 190136

Group	Median AUC from Day 91–112 (min, max)	*P*-value (vs challenge control)	*P*-value (vs revaccination)
Challenge control	65.4 (61.0, 79.2)	N/A	< 0.0001
Revaccination	20.2 (19.2, 22.9)	< 0.0001	N/A
Single vaccination	34.9 (24.5, 41.4)	< 0.0001	0.0029

Challenge control group (*n* = 16); Revaccination group (*n* = 16); Single vaccination group (*n* = 16)

Viral RNA load and AUC were assessed using RT-qPCR

*AUC* area under the curve, *N/A* not applicable, *PRRSV* Porcine reproductive and respiratory syndrome virus, *RT-qPCR* reverse transcription quantitative polymerase chain reaction

### Gilt PRRS serology

PRRS serology results from Day 0 to Day 91 are shown in Table 2. All gilts were PRRSV seronegative at the start of the study, as indicated by ELISA. As expected, all challenge control gilts were PRRSV seronegative up to Day 91 (day of challenge). Gilts in the revaccination group were all seronegative on Day 0 (day of first vaccination), 25% were PRRS seropositive at Day 14, and 69% were seropositive at Day 56 (day of second vaccination), consistent with a serological response to the first vaccination. The proportion of seropositive gilts did not increase further following the second vaccination. In the single-vaccination group, all gilts were PRRSV seronegative up to Day 56 (day of vaccination) and 69% were PRRSV seropositive by Day 70, consistent with a serological response to vaccination. From Day 77, a numerically higher percentage of gilts in the single-vaccination group (94%) were seropositive compared with the revaccination group (69%), and this difference was statistically significant at Day 91 (94% vs 56%, respectively; *P* = 0.0373).

**Table 2:** Percentage of gilts who were seropositive for PRRSV based on ELISA

Day	Group	% seropositive (95% CI)	P-value (vs challenge control)	*P*-value (vs revaccination)
0	Challenge control	0	–	–
	Revaccination	0	–	–
	Single vaccination	0	–	–
14	Challenge control	0 (0.0, 20.6)	N/A	
	Revaccination	25 (7.3, 52.4)	0.101	N/A
	Single vaccination	0 (0.0, 20.6)	ND	0.101
21	Challenge control	0 (0.0, 20.6)	N/A	
	Revaccination	56 (29.9, 80.2)	0.0008	N/A
	Single vaccination	0 (0.0, 20.6)	ND	0.0008
56	Challenge control	0 (0.0, 20.6)	N/A	
	Revaccination	69 (41.3, 89.0)	0.0001	N/A
	Single vaccination	0 (0.0, 20.6)	ND	0.0001
70	Challenge control	0 (0.0, 20.6)	N/A	
	Revaccination	69 (41.3, 89.0)	0.0001	N/A
	Single vaccination	69 (41.3, 89.0)	0.0001	1.0000
77	Challenge control	0 (0.0, 20.6)	N/A	
	Revaccination	69 (41.3, 89.0)	0.0001	N/A
	Single vaccination	94 (69.8, 99.8)	< 0.0001	0.172
91	Challenge control	0 (0.0, 20.6)	N/A	
	Revaccination	56 (29.9, 80.2)	0.0008	N/A
	Single vaccination	94 (69.8, 99.8)	< 0.0001	0.0373

Challenge control group (*n* = 16); Revaccination group (*n* = 16); Single-vaccination group (*n* = 16)

PRRS serology was assessed using ELISA

*CI* confidence interval, *ELISA* enzyme-linked immunosorbent assay, *N/A* not applicable, *ND* not determined, *PRRSV* Porcine reproductive and respiratory syndrome virus

### Clinical signs

Three of the 16 gilts (19%) in both the challenge control and revaccination groups, and two of 16 gilts (13%) in the single-vaccination group, exhibited abnormal clinical signs such as skin lesions, lameness, and thinness. In three challenge control gilts, these abnormal clinical observations were exhibited before challenge on Day 91, and one of the single-vaccination gilts also exhibited clinical signs before receiving a vaccination on Day 56, all clinical signs were recorded before challenge. These clinical signs were determined by the study investigator as not interfering with animal welfare and to be unrelated to PRRS or study medication.

## Discussion

The study aimed to determine if a revaccination schedule with ReproCyc® PRRS EU has a vaccine efficacy at least equivalent (quantitatively and qualitatively) to that seen with a single-vaccination scheme. All gilts entering the study were free from PRRSV as demonstrated by the absence of PRRSV RNA in their serum followed by PRRS seronegative ELISA test results on Day 0. In addition, RT-qPCR and serology results indicated all gilts were free from PRRSV from Day 0 to Day 91.. These data confirm that no extraneous PRRSV exposure or cross-contamination among treatment and control groups occurred up to the point of challenge on Day 91, validating subsequent findings of this study. Viremia in adult animals is usually short-lasting; therefore, the lack of observed vaccine-induced viremia prior to challenge may be due to resolution of viremia before the initial blood samples were taken 14 days post-vaccination.

The study found that both single vaccination and revaccination with ReproCyc® PRRS EU significantly reduced PRRSV viremia post-challenge compared with non-vaccinated challenged controls. However, the revaccination group had a significantly lower viral RNA load and fewer PRRSV RNA-positive gilts on Day 101 compared with the single vaccination group. Furthermore, at all timepoints following challenge, both the mean viral RNA loads and the percentage of PRRSV RNA-positive gilts were numerically higher in the single-vaccination group than in the revaccination group. In addition, the revaccination group had a significantly lower viremia AUC_91–112_ compared with the single-vaccination group.

These findings suggest that previous vaccination with ReproCyc® PRRS EU increases the effectiveness of subsequent vaccination with the same product, and revaccination may be slightly more effective against PRRSV post-challenge than a single-vaccination schedule. Although there was no correlation between the PRRS seroconversion and viremia reductions across the vaccination schemes, the consistently greater reduction in viremia observed with revaccination compared with single vaccination suggests an element of protective immunity provided by the revaccination schedule, possibly linked to the presence of memory T cells.

Seroconversion of gilts was not complete in either the single-vaccination or revaccination group. Furthermore, the seroconversion rate was particularly low in the revaccination group (56% seropositive on Day 91) and did not increase following the second vaccination, despite the greater reduction in viremia observed with revaccination compared with single vaccination. The authors do not have an explanation for this unexpected finding, which differs from other studies that have shown consistent seroconversion following vaccination [25, 26]. These observations suggest that seroconversion is not an indicative measure of protection in adult animals. Some animals did not test positive by either RT-qPCR or ELISA and showed the same level of protection upon challenge compared with animals that had seroconverted. Incomplete seroconversion alongside full protection from PRRS has already been observed in other studies [27] and most likely reflects variability in immune responses between animals.

Previous studies demonstrated that a single-dose of ReproCyc® PRRS EU was successful in protecting piglets [22, 24], bred gilts [21], and farrowing gilts [23] from PRRS. Vaccinating gilts helps prevent viral shedding and reduces both horizontal (sow to sow) and vertical PRRSV transmission (sow to piglet). This study suggests that a single shot of vaccination is sufficient to protect gilts based on reduced viremia, and thereby likely to reduce the risk of transmission as well. It also showed that revaccinating non-pregnant gilts reduced challenge-induced viremia compared with single vaccination; this effect could reduce shedding, thus decreasing airborne levels of PRRSV, preventing infection of pregnant gilts and consequently reducing transmission of PRRSV to unborn piglets. Given the duration of immunity defined in other studies [28], we estimate that a revaccination schedule of every 3 to 4 months would be sufficient to avoid gaps in protection from PRRS.

Due to the wide heterology of PRRSV field strains [29] the level of protection provided by a single vaccine strain may differ from farm to farm. Although genetic distance in the ORF5 gene locus is not a stoichiometric indicator of the likelihood of protection, the protection demonstrated in this study with the chosen genetic distance of less than 87% homology between the vaccine and challenge should provide a good level of assurance that the vaccine could protect against heterologous PRRSV strains. As in previous cases, this should also be verified in field studies [27].

While this study was designed to look at the clinical outcomes of revaccination related to PRRS rather than on immunological responses to recurring vaccination, there was no evidence of detrimental immunologic effects (such as immune reactions to the vaccine composition) with the revaccination schedule. ReproCyc® PRRS EU was well tolerated; two or three gilts per group showed clinical signs that are typically associated with gilts housed in groups, with some developing before treatment with study medication or in the control group. These signs were unlikely to be attributable to vaccination and more associated with gilts establishing a hierarchy that involved biting, scratching, and exclusion of pen mates from the feeder (hence leading to some thinner gilts compared with pen mates). Together with the efficacy data, this supports the feasibility of recurring vaccination of sows and mass vaccination in a whole herd approach.

Further studies that include a greater number of animals are needed to confirm the benefit of revaccination against PRRSV.

## Conclusion

This study shows that gilts vaccinated with ReproCyc® PRRS EU, either using a revaccination or single-vaccination schedule, demonstrate significant protection from viremia after challenge with PRRSV Strain 190,136 compared with unvaccinated challenge control gilts. Furthermore, viremia levels after challenge were significantly lower in the revaccination group than in the single-vaccination group based on median viral RNA load AUC_91–112_, suggesting that revaccinated gilts had better protection from viral infection than gilts who received a single vaccination. ReproCyc® PRRS EU was also well tolerated and no safety concerns were identified.

## Methods

### Animals

Animals (48 commercial crossbred gilts) were provided by the Wilson Prairie View Farms, N5627 Highway DD, Burlington, WI 53105, USA, and each female was individually ear-tagged with a unique number. All gilts were healthy (as determined by observation), PRRS seronegative (as determined by ELISA), and were not pregnant on Day 0.

Gilts were housed in separate, but uniform rooms from Day − 10 to study completion. Each room consisted of four pens, with four gilts per pen and was biohazard level 2 compliant, hepafiltered, mechanically ventilated with thermostatically regulated temperature control. Treatment group isolation was necessary to avoid cross-contamination between groups.

Gilts were fed a commercially prepared, non-medicated gestation ration of solid feed (Heart of Iowa Cooperative, Roland, IA, USA), which was stored in bags, free from vermin, and given in quantities appropriate for each gilt’s size, age, and condition. Water was available ad libitum.

### PRRSV immunization and challenge inoculum

The control product (lyophilized placebo, Boehringer Ingelheim Animal Health, Inc., St Joseph, USA; lot N240-191-062409) and the investigational veterinary product (IVP; ReproCyc® PRRS EU, Boehringer Ingelheim Animal Health, Inc., USA; lot 390-007A), which was at a concentration of 1 × 10^4^ 50% tissue infectious dose (TCID_50_)/2 mL, were administered by intramuscular (IM) injection, using sterile, appropriately sized Luerlock syringes and sterile 20 g × 1 in. (2.54 cm) needles. Animals were injected in the neck, midway between the point of the shoulder and the base of the ear.

All gilts were challenged with PRRSV strain 190,136 (Boehringer Ingelheim Vetmedica, Inc., USA), which was derived from lung tissue of a newborn piglet during an outbreak in Germany in April 2004 and exhibits less than 87% genetic identity to ReproCyc® PRRS EU within the complete genome (or 88% based on ORF5/ORF7 data). Challenge was intranasally (2.0 mL per nostril) and intramuscularly (2.0 mL injection to the back of the neck) using a target titer of 1 × 10^5.9^ TCID_50_/6 mL.

### Experimental design

This was a blinded revaccination-challenge laboratory efficacy study consisting of three groups: challenge control group (*n* = 16), revaccination group (*n* = 16) and single-vaccination group (*n* = 16). In the challenge control group, a 2.0 mL injection of the control product was given on Day 0 (right neck) and Day 56 (left neck). In the revaccination group, the IVP was given on Day 0 (right neck) and Day 56 (left neck). In the single-vaccination group, a 2.0 mL injection of the control product was given on Day 0 (right neck) and a 2.0 mL of the IVP on Day 56 (left neck). All gilts were challenged on Day 91. The Study Investigator and designees were blinded to group assignments and laboratory personnel were blinded to the treatment each gilt received. In addition, a person not collecting data administered the injections of study products. In this study, 8 weeks between vaccinations was utilized as this allowed for adequate time for immunity to be developed after the first vaccination before revaccination occurred.

### Sample analysis and assessment of viremia and serology

Blood samples were collected prior to animal enrolment and on Days 0, 14, 21, 56, 70, 77, 91, 94, 98, 101, 105, and 112. After drawing, blood samples were allowed to clot at room temperature, were centrifuged and the serum was harvested. Serum samples were held at − 20 °C for serology testing and − 70 °C (± 10 °C) for RT-qPCR testing.

### PRRS serology

For ELISA, the IDDEX PRRS X3 test was used following the manufacturer’s instructions (HerdChek* Porcine Reproductive and Respiratory Syndrome Antibody Test Kit X3 – IDEXX Laboratories Inc., Westbrook, ME, USA). Results were reported as negative (ELISA sample to positive [S/P] ratio of < 0.4) or positive (ELISA S/P ratio of ≥0.4).

### PRRS serum RT-qPCR

TaqMan RT-qPCR targeting the viral open reading frame (ORF) 7 was used to detect viral RNA. Results were reported as log_10_ genome equivalent (GE)/mL. A RT-qPCR result of ND (not detected) was assigned a value of 0 log_10_ GE/mL and a positive but unquantifiable RT-qPCR result was assigned a log_10_ value of 3.0 GE/mL (for statistical purposes only).

### Assessment of clinical signs

Gilts were observed once daily from Day − 1 to Day 112 for abnormal health by the Study Investigator or designees. An adverse event was defined as any observation that was unfavorable or unintended that occurred after the use of the IVP, irrespective of causality.

### Statistical analysis

Gilts were randomly assigned to one of the groups prior to Day − 1. Each group included 16 animals, which was expected to provide approximately 80% power to detect a difference of 20 percentage points for median viral load between the treatment and control group for a two-sided test using an alpha value of 0.05 post-challenge.

Data were summarized using descriptive statistics with a 95% confidence interval. All tests on differences were designed as two-sided tests using an alpha value of 5%. Statistical analyses were performed using SAS software release 8.2 (SAS Institute Inc., 2001, Cary, North Carolina, USA).

## References

[1] Chung WB, Lin MW, Chang WF, Hsu M, Yang PC (1997). Persistence of porcine reproductive and respiratory syndrome virus in intensive farrow-to-finish pig herds. Can J Vet Res.

[2] Done SH, Paton DJ, White ME (1996). Porcine reproductive and respiratory syndrome (PRRS): a review, with emphasis on pathological, virological and diagnostic aspects. Br Vet J.

[3] World Organisation for Animal Health (OiE) (2008). Report of the OIE ad hoc group on porcine reproductive respiratory syndrome.

[4] Wills RW, Zimmerman JJ, Yoon KJ, Swenson SL, McGinley MJ, Hill HT, Platt KB, Christopher-Hennings J, Nelson EA (1997). Porcine reproductive and respiratory syndrome virus: a persistent infection. Vet Microbiol.

[5] Charerntantanakul W (2012). Porcine reproductive and respiratory syndrome virus vaccines: immunogenicity, efficacy and safety aspects. World J Virol.

[6] Kuhn JH, Lauck M, Bailey AL, Shchetinin AM, Vishnevskaya TV, Bao Y, Ng TF, LeBreton M, Schneider BS, Gillis A (2016). Reorganization and expansion of the nidoviral family Arteriviridae. Arch Virol.

[7] Yaeger M, Prieve T, Collins J, Christopher-Hennings J, Nelson E, Benfeld D (1993). Evidence for the transmission of porcine reproductive and respiratory syndrome (PRRS) virus in boar semen. Swine Health Prod.

[8] Yoon IJ, Joo HS, Christianson WT, Morrison RB, Dial GD. Persistent and contact infection in nursery pigs experimentally infected with porcine reproductive and respiratory syndrome (PRRS) virus. Swine Health Prod. 1993;1(4):5-8.

[9] Rossow KD, Bautista EM, Goyal SM, Molitor TW, Murtaugh MP, Morrison RB, Benfield DA, Collins JE (1994). Experimental porcine reproductive and respiratory syndrome virus infection in one-, four-, and 10-week-old pigs. J Vet Diagn Investig.

[10] Collins JE, Benfield DA, Christianson WT, Harris L, Hennings JC, Shaw DP, Goyal SM, McCullough S, Morrison RB, Joo HS (1992). Isolation of swine infertility and respiratory syndrome virus (isolate ATCC VR-2332) in North America and experimental reproduction of the disease in gnotobiotic pigs. J Vet Diagn Investig.

[11] Corzo CA, Mondaca E, Wayne S, Torremorell M, Dee S, Davies P, Morrison RB (2010). Control and elimination of porcine reproductive and respiratory syndrome virus. Virus Res.

[12] Charpin C, Mahe S, Keranflec'h A, Belloc C, Cariolet R, Le Potier MF, Rose N (2012). Infectiousness of pigs infected by the porcine reproductive and respiratory syndrome virus (PRRSV) is time-dependent. Vet Res.

[13] Nodelijk G, de Jong MC, Van Nes A, Vernooy JC, Van Leengoed LA, Pol JM, Verheijden JH (2000). Introduction, persistence and fade-out of porcine reproductive and respiratory syndrome virus in a Dutch breeding herd: a mathematical analysis. Epidemiol Infect.

[14] Rowland RR, Lunney J, Dekkers J (2012). Control of porcine reproductive and respiratory syndrome (PRRS) through genetic improvements in disease resistance and tolerance. Front Genet.

[15] Scortti M, Prieto C, Simarro I, Castro JM (2006). Reproductive performance of gilts following vaccination and subsequent heterologous challenge with European strains of porcine reproductive and respiratory syndrome virus. Theriogenology.

[16] Rowland RR (2010). The interaction between PRRSV and the late gestation pig fetus. Virus Res.

[17] Cano JP, Dee SA, Murtaugh MP, Pijoan C (2007). Impact of a modified-live porcine reproductive and respiratory syndrome virus vaccine intervention on a population of pigs infected with a heterologous isolate. Vaccine.

[18] Cano JP, Dee SA, Murtaugh MP, Trincado CA, Pijoan CB (2007). Effect of vaccination with a modified-live porcine reproductive and respiratory syndrome virus vaccine on dynamics of homologous viral infection in pigs. Am J Vet Res.

[19] Kimman TG, Cornelissen LA, Moormann RJ, Rebel JM, Stockhofe-Zurwieden N (2009). Challenges for porcine reproductive and respiratory syndrome virus (PRRSV) vaccinology. Vaccine.

[20] Huang YW, Meng XJ (2010). Novel strategies and approaches to develop the next generation of vaccines against porcine reproductive and respiratory syndrome virus (PRRSV). Virus Res.

[21] Piontkowski MD, Kroll J, Orveillon FX, Kraft C, Coll T (2016). Safety and efficacy of a novel European vaccine for porcine reproductive and respiratory virus in bred gilts. Can J Vet Res.

[22] Piontkowski M, Kroll J, Kraft C, Coll T (2016). Safety and early onset of immunity with a novel European porcine reproductive and respiratory syndrome virus vaccine in young piglets. Can J Vet Res.

[23] Stadler J, Zoels S, Eddicks M, Kraft C, Ritzmann M, Ladinig A (2016). Assessment of safety and reproductive performance after vaccination with a modified live-virus PRRS genotype 1 vaccine in pregnant sows at various stages of gestation. Vaccine.

[24] Cano G, Cavalcanti MO, Orveillon FX, Kroll J, Gomez-Duran O, Morillo A, Kraft C (2016). Production results from piglets vaccinated in a field study in Spain with a type 1 porcine respiratory and reproductive virus modified live vaccine. Porcine Health Manag.

[25] Zuckermann FA, Garcia EA, Luque ID, Christopher-Hennings J, Doster A, Brito M, Osorio F (2007). Assessment of the efficacy of commercial porcine reproductive and respiratory syndrome virus (PRRSV) vaccines based on measurement of serologic response, frequency of gamma-IFN-producing cells and virological parameters of protection upon challenge. Vet Microbiol.

[26] Klinge KL, Vaughn EM, Roof MB, Bautista EM, Murtaugh MP (2009). Age-dependent resistance to porcine reproductive and respiratory syndrome virus replication in swine. Virol J.

[27] Balka G, Dreckmann K, Papp G, Kraft C (2016). Vaccination of piglets at 2 and 3 weeks of age with Ingelvac PRRSFLEX(R) EU provides protection against heterologous field challenge in the face of homologous maternally derived antibodies. Porcine Health Manag.

[28] Summary of product characteristics: ReproCyc PRRS EU lyophilisate and solvent for suspension for injection for pigs. In: Health products regulatory authority; 2015. https://imedi.co.uk/reprocyc-prrs-eu-lyophilisate-and-solvent-for-suspension-for-injection-for-pigs.

[29] Stadejek T, Stankevicius A, Murtaugh MP, Oleksiewicz MB (2013). Molecular evolution of PRRSV in Europe: current state of play. Vet Microbiol.

